# Mental health disorders and quality of life among Saudi women who have experienced domestic violence

**DOI:** 10.3389/fpubh.2025.1568733

**Published:** 2025-05-30

**Authors:** Hend Faye AL-shahrani, Mohammad Ahmed Hammad

**Affiliations:** ^1^Department of Social Planning, College of Humanities and Social Sciences, Princess Noura bint Abdul Rahman University, Riyadh, Saudi Arabia; ^2^College of Education, Najran University, Najran, Saudi Arabia; ^3^College of Education, Assiut University, Assiut, Egypt

**Keywords:** domestic violence, mental health, anxiety, depression, quality of life, Saudi women

## Abstract

**Objective:**

The present cross-sectional study aimed to examine the nature of domestic violence among women in Saudi Arabia, to explore its association with mental health disorders such as depression and anxiety, and to examine its impact on victims’ quality of life.

**Methods:**

This cross-sectional descriptive study was conducted in Riyadh region, Saudi Arabia. Data were collected from 387 women from the Riyadh region who had experienced domestic violence (Mean age = 32.47 years; SD = 4.26 years) using a systematic random sampling method. Quantitative data were gathered using the Domestic Violence Scale, the Depression Scale (CES-D-10; Andresen et al., 1994), the General Anxiety Scale (GAD-7; Spitzer et al., 2006), and the Quality of Life Scale (WHOQOL-BREF; Group, 1998).

**Results:**

The results revealed that 36.68% of the participants experienced moderate to severe levels of domestic violence. Psychological violence was more common than physical violence, while sexual violence was the least prevalent. Additionally, employed, more educated, and older women tended to experience lower levels of domestic violence as compared to their counterparts. Moreover, women who had experienced domestic violence exhibited higher severity of depression and anxiety symptoms. A linear regression analysis indicated that domestic violence was a powerful predictor of depression, anxiety, and poor quality of life in this sample.

**Conclusion:**

These findings highlight the need to further examine the adverse effects of domestic violence on women and their children, and to prioritize mental health and quality of life interventions for such women. The insights gained from this study could also inform the design of programs aimed at preventing domestic violence.

## Introduction

Domestic violence (also known as “intimate partner violence” or “domestic abuse”) entails behaviors that seek to exercise control over one’s partner (1), including but not limited to “the intentional use of physical force or force against oneself, or another person, group or community in a manner that causes physical harm, psychological harm, death, developmental problems, or deficiency” (2). Such violent acts are mostly committed in the family environment and they endanger a person’s body, life, psychological integrity or freedom by the use of force or coercion (3). It is widely recognized as a serious public health problem affecting victims’ well-being (4). Domestic violence adversely affects the well-being of victims and their families, with lasting consequences for both physical and mental health, even after the violence has ceased (5). Furthermore, Malik et al. (6) indicates that domestic violence can significantly harm the physical and mental health of abused women. Additionally, it undermines their social, economic, spiritual, and emotional well-being, ultimately impacting the broader community. This issue is recognized as a major contributor to the decline of women’s mental and physical health (7).

Domestic violence is a global phenomenon, with psychological abuse being the most common form (8, 9). However, victims may be subjected to one or more, or sometimes, all forms of violence within the family (10, 11). Physical violence involves using brute force with the intention to intimidate and punish. Sexual violence can be defined as using unwanted sexual advances to threaten, intimidate, and control another person (12). Psychological violence refers to verbal insults and threats to intimidate, control, and punish a person (13). Economic violence, conversely, is defined as withholding money and resources to control another person (14). Furthermore, in a series of population-based studies from Turkiye, Alkan et al. (15, 16) reported that victims who had experienced one type of domestic violence were more likely to experience the other types as well.

While victims of domestic abuse may include children and other members of a household, the intimate partner is often the primary victim (17), with women being more vulnerable to experiencing such abuse (13). This may be attributed to prevalent gender inequality and socio-cultural norms and structural factors that perpetuate male dominance and/or condone the use of violence to assert one’s power (18), age, marital status, lower socioeconomic status, religion, occupation, history of experiencing or witnessing violence, etc. (17, 19–21).

### Literature review

Domestic violence has a variety of physical and mental health consequences. Victims often experience anxiety, depression, and an increased risk of suicide. Exposure to such traumatic events can result in stress, fear, and isolation, which can further contribute to feelings of depression, anxiety, poor quality of life and suicidal thoughts or behaviors., Studies have reported a high incidence of depression, anxiety, Post-Traumatic Stress Disorder (PTSD), and poor quality of life among victims (6, 13, 20, 22–28). Physical violence, often accompanied by psychological and sexual abuse, has been reported to lead to poor mental health (29).Tolman and Rosen (30) reported that 9.2 to 13.4% of female welfare recipients who experienced intimate partner violence had generalized anxiety disorder, 31.3 to 44.6% had depression, while 17.6 to 38.4% had PTSD. Similarly, Hegarty, O’Doherty (31) found that experiencing increasingly severe violence is associated with worse social adjustment and higher levels of depression, anxiety, and PTSD. This was also confirmed by Ferrari et al. (32), who reported increased mental health problems after experiencing physical violence. Beck et al. (29) reported average prevalence rates of 34.4% for anxiety, 18.8% for depression, and 76.3% for diagnoses of other co-existing mental illnesses. Thus, women who have experienced domestic violence often exhibit significant mental health difficulties involving multiple diagnoses. Other studies have reported that 40% of women who have experienced domestic violence reported clinical levels of distress, at least 70% reported symptoms of depression or anxiety, and 77% had symptoms of PTSD (33). As compared to the general population, survivors of domestic violence reported higher levels of violence and depression when they first contacted therapeutic services (34). However, these levels decrease over time, regardless of whether women receive treatment (32, 35). Age can be a confounding variable in the relationship between exposure to domestic violence and mental health. Although younger women may be at higher risk of current violence, older women have more life experience, which helps them to contain various situations, thus reducing the severity of the violence experienced (32). Higher level of education and job status are likely to protect against exposure to domestic violence. Therefore, socioeconomic status should be considered when analyzing the relationship between domestic violence exposure and mental health.

While domestic violence is a universal concern, its manifestation and impact vary across cultures and regions (36). Studies from different regions of the world report that 4 to 49% of women have experienced physical violence from their husbands at some point during their lifetime (37). A multicenter study, conducted by the World Health Organization, with 24,000 women across 10 countries revealed a lifetime prevalence of 13–61% for physical violence, 20–75% for emotional violence, and 6–59% for sexual violence (37). The 2014 Violence Against Women and the World in Reality report stated that one in three women (about 62 million) aged over 15 years had experienced violence, and that 8% of those had experienced physical and sexual violence within the past twelve months (2). A systematic review of 74 studies on intimate partner violence against women from 11 Arab countries (including Saudi Arabia) reported a prevalence of 6–59% for physical violence, 3–40% for sexual violence, and 5–91% for emotional/psychological violence (38). A UN report on violence against women in Arab countries emphasized that intimate partner violence is not just a human rights issue but that it also endangers women’s overall wellbeing (39). Further addressing factors that render women more vulnerable to violence in Arab countries, the report highlighted sociocultural factors prevalent in the region, such as traditional male dominant gender norms, poor legal measures against perpetrators of violent acts against women, lack of women’s participation in social, political, and economic spheres (39). Specifically in Saudi Arabia, traditional family values, gender inequity, and varying interpretations of religious tenets (which are perceived to justify women’s abuse) may contribute to the tolerance of and/or low reporting of domestic violence against women (40).

## The current study

Interestingly, although the issue of domestic violence came to the forefront about 39 years ago, most studies on domestic violence against women in Saudi Arabia were conducted only in the last ten to fifteen years (25, 27, 41–45). With limited research in this area, there is a compelling need to explore the epidemiological dimensions of domestic violence among women in Saudi Arabia (46). Globally, domestic violence statistics feature the urgency of understanding and mitigating its impact. The occurrence of physical, emotional, and sexual violence against women requires an attentive investigation, particularly in regions like Saudi Arabia, where research gaps continue to exist (1, 2).

The Saudi culture and society poses certain barriers that limits conversations on domestic violence in the personal, social, political, and even academic spheres. The few studies that have examined the prevalence of domestic violence in Saudi Arabia report a lifetime prevalence ranging from 35 to 45% (42, 47, 48). Similarly, a systematic review of 11 studies conducted in six cities in Saudi Arabia reported a lifetime prevalence of 39.3–44.5% (49). Furthermore, the mental health outcomes of Saudi women who have experienced domestic violence are yet to be explored. In general, women in Saudi Arabia hold a low social status, often limited by legislative, social, educational, and occupational constraints (50). This gender inequity, narrow interpretations of Islamic laws and social norms have a negative impact on the health and well-being of women (51). However, specifically in the case of women who have experienced domestic violence, help-seeking behaviors may be low owing to these sociocultural factors (40, 52). Recognizing the far-reaching implications of domestic violence on the mental health and overall quality of life of victims, this study sought to contribute to the broader dialog surrounding the importance of addressing this societal concern. We aim to offer insights that extend beyond statistical figures, providing a deeper understanding of what women face in Saudi Arabia. This foundational knowledge is crucial for formulating effective strategies to alleviate the burden of domestic violence on women’s lives.

In a nutshell, the current study addresses the gap in understanding the nature, influencing factors, and impact of domestic violence experienced by women in Riyadh region, Saudi Arabia, to inform targeted interventions for victims. Specifically, we aimed to (1) examine the nature of domestic violence experienced by our sample of women from Riyadh region, Saudi Arabia; (2) investigate the association of domestic violence with mental health outcomes (viz., depression and anxiety) and quality of life while accounting for important potential confounding factors, such as age, education, job status, and health status. Three research questions were posed to achieve these objectives:

1. What is the nature of domestic violence experienced by a sample of women from the Riyadh region, Saudi Arabia?
2. Does domestic violence predict anxiety, depression, and quality of life in a sample of women from the Riyadh region, Saudi Arabia?
3. Does domestic violence vary based on other variables such as age, education, employment status, and health status?

## Research methodology

### Participants

This cross-sectional descriptive study included 387 women from Riyadh region Saudi Arabia, aged 18–60 years (Mean age = 38.56 years; SD age = 4.24 years), who had experienced domestic violence at least once in the past. The sample size for this study was determined using G*Power version 3.1.9.7 with 0.05 *α* error probability and 80% power. The estimated sample size of 387 women was deemed appropriate to achieve statistical validity. Advertisements were posted on social media, inviting Saudi women to participate in the study. From among those who expressed interest, purposive sampling was used to select participants who fit the following selection criteria: having experienced domestic violence at least once in their lifetime, being aged 18–60 years, being able to read Arabic, being a resident of southern Saudi Arabia, not having any intellectual or sensory disabilities or any other problems that prevented them from completing the study questionnaire.

### Measures

Data were collected using a self-report online questionnaire that comprised two sections. The first section included questions on the demographic and social characteristics of participants (age, education, job status, and health status). Age was classified into three categories (18–29 years, 30–44 years, and over 45-years). Educational level was categorized into three groups (high education “graduate and above”, medium education “up to secondary education,” not educated). Job status and health status were both divided into two categories (employed and not employed, and good and sick, respectively).

The second section included the standardized tools used to assess the four core study variables of domestic violence, depression, anxiety, and quality of life. Each tool has been described in the sub-sections that follow.

### Domestic violence questionnaire

This instrument evaluates domestic violence through 15 items distributed across four domains or types. The tool first defines the four types to participants. Specifically, physical violence is described as threatening with sharp objects, throwing objects, or hitting and pushing. Verbal violence is described as insults and humiliation of women or their families. Psychological violence is described as threatening to marry another woman, threatening divorce, doubting, screaming, ignoring the wife, and checking her phone. Sexual violence is described as forcing the wife to have sex without her consent, the desire to have sex during menstruation, unusual sexual behavior without the wife’s consent, reluctance or distance from having sex with the wife, and forcing the partner to watch vulgar movies. Items in each domain measured the level to which the respondent experienced that type of domestic violence, using a 5-point Likert scale ranging from 1, indicating “not at all” exposed to the situation, to 5, indicating “very much” exposed to the situation. Scale scores range from 15 to 75, with higher scores indicating higher levels of domestic violence experienced.

The degree of domestic violence is divided into three levels (high, medium, and low) using the following equation:


$$\frac{\text{highest response}-\text{lowest response}}{\text{number of categories}}=\frac{5-1}{3}=\frac{4}{3}=1.33$$

Accordingly, scale scores <2.33 denote low domestic violence, scores between 2.34–3.67 denote moderate domestic violence, and scores > 3.68 demote high domestic violence. The overall internal consistency reliability scores for each domain ranged from 0.56 to 0.68. The Cronbach’s alpha for individual items ranged from 0.74 to 0.87, and that for the overall scale score was 0.77, indicating optimal internal consistency. The coefficient of split-half reliability was 0.86, indicating acceptable psychometric properties for use in the present study.

### Generalized anxiety disorder questionnaire (GAD-7)

The GAD-7 scale was developed as a short tool to quickly assess the severity of generalized anxiety disorder (53). The questionnaire consists of seven items asking respondents how often they have felt upset in the past two weeks, with each item representing one of the seven main symptoms of generalized anxiety disorder listed in the DSM-IV. Response options range from 0 to 3 (“not at all” to “almost every day”), with overall scores ranging from 0 to 21. Scores of 5, 10, and 15 are used as cut-offs to indicate mild, moderate, and severe anxiety symptoms, respectively. The scale showed good internal consistency (Cronbach *α* = 0.92) and reliability (correlation within the category = 0.83) in the original study (53). Later, several studies showed high internal consistency, test–retest reliability, and convergent validity [e.g., García-Campayo et al. (54) and Zhong et al. (55)]. The current study showed good internal consistency (Cronbach *α* = 0.77).

### Centre for epidemiological studies depression scale (CES-D-10)

This scale consists of ten items measuring depression symptoms experienced during the week preceding the assessment (56). It includes three items assessing depressive affect, five assessing physical symptoms, and two evaluating positive affect (which are reverse-scored). Each item is rated on a four-point Likert scale ranging from “rare or non-existent” (0) to “all the time” (3). Total scores range from 0 to 30, and higher scores indicate higher severity of depressive symptoms. In the original study, CES-D-10 showed good internal consistency and reliability (56). The Arabic version of CES-D-10 (57) exhibited high internal consistency (Cronbach’s *α* of ≥. 0.88) and has being utilized in prior Saudi studies (45, 58). The tool also showed good internal consistency in the present study (Cronbach *α* = 0.81).

### WHO quality of life-BREF (WHOQOL-BREF)

The abbreviated WHOQOL-BREF scale contains 24 items (59) assessing quality of life in four main areas: physical ability (7 items), psychological well-being (6 items), social relations (3 items), and environment (8 items). All items are rated on a 5-point Likert scale with total scores ranging from 25 to 125 points. Higher scores represent greater quality of life. The Arabic version of WHOQOL-BREF (60) has exhibited high internal consistency (Cronbach’s *α* of ≥0.70) in previous samples of Saudi residents (41, 61). The tool showed good internal consistency in the present study (Cronbach *α* = 0.79).

### Data collection procedure

Advertisements were posted on social media, inviting Saudi women to participate in the study, with a link that provided complete information about the study. Participants were informed that completion of the questionnaire would indicate their consent to voluntary participation. It was clarified that the data collected would remain strictly confidential and would only be used for research purposes. Before starting the study, we obtained the ethical approval of the Deanship of Scientific Research at Princess Noura bint Abdul Rahman University Riyadh, Kingdom of Saudi Arabia (PNURSP2025R707). The Ethics Committee of Scientific Research at the university also approved the study questionnaire (Domestic Violence Questionnaire) after completing validity and reliability procedures and approved its use with the study sample. In addition, all study procedures complied with those outlined in the Declaration of Helsinki. The initial sample comprised 400 participants. Thirteen participants were excluded due to incomplete data. The final sample of 387 participants represented women from southern Saudi Arabia who had experienced domestic violence at least once in their lifetime. The data collection took about two months.

### Data analysis

All statistical analyses were conducted using SPSS, version 21. Frequency and percentage were used to examine the nature of domestic violence experienced by the present sample. In addition, means and standard deviations were used, as appropriate. Bivariate comparisons of domestic violence scores by demographic characteristics was conducted using t-tests and one-way ANOVA, as appropriate. Finally, linear regression analysis was conducted to examine the association of domestic violence scores with depression, anxiety, and quality of life, while controlling for demographic variables that exhibited significant group differences in the bivariate analyses. Prior to running inferential statistics, all relevant test assumptions were checked, including normal distribution and homoscedasticity.

## Results

The prevalence of domestic violence among the present sample of Saudi women is presented in Table 1, with severity levels of domestic violence being categorized as low, moderate, and severe. In terms of severity level, majority of the participants (63.30%) experienced low levels of domestic violence, followed by moderate (31.26%) and severe levels of domestic violence (5.42%).

**Table 1:** Distribution of types of domestic violence among Saudi women by severity.

	Low *N* (%)	Moderate *N* (%)	Severe *N* (%)
Psychological abuse	172 (44.44)	169 (43.66)	46 (11.88)
Physical abuse	229 (59.17)	147 (37.98)	11 (2.84)
Sexual abuse	334 (84.3)	47 (11.9)	6 (1.5)
Domestic violence	245 (63.30)	121 (31.26)	21 (5.42)

Table 1 shows the distribution of each type of domestic violence in the current sample, classified into low, moderate, and severe levels of domestic violence.

Note that over half (55.54%) of the participants experienced moderate to severe levels of psychological abuse, while the comparative incidence of similar levels of physical and sexual abuse was 40.82 and 13.4%, respectively. Table 2 summarizes the results of one-way ANOVA conducted to examine differences in domestic violence based on educational level and age. The results revealed statistically significant differences in domestic violence for both variables (*p* < 0.01).

**Table 2:** ANOVA comparing domestic violence scores by educational level and age.

Source	Type III sum of squares	Push	Mean square	*F*	Sig.
Educational level	839.687	2	419.843	24.584	0.014
Age	147.954	2	73.977	4.332	0.000

To further assess the nature of these differences, Scheffe test was used for post-hoc analyses of differences by educational level (Table 3) and age (Table 4). It was observed that domestic violence scores were the lowest for participants with high education, followed by those with medium and no education, respectively. Thus, higher the education, the lower was the severity of domestic violence experienced.

**Table 3:** Scheffe test comparison of differences in domestic violence scores based on educational level.

(I) Educational level	(J) Educational level	Mean difference (I-J)	Std. error	Sig.	95% Confidence interval	
					Lower bound	Upper bound
High education	Medium education	−8.53^*^	0.47	0.000	−9.69	−7.36
	Not educated	−14.85^*^	0.58	0.000	−16.29	−13.40
Medium education	High education	8.53^*^	0.47	0.000	7.36	9.69
	Not educated	−6.32^*^	0.62	0.000	−7.86	−4.77
Not educated	High education	* 14.85^*^	0.58	0.000	13.40	16.29
	Medium education	6.32	0.62	0.000	4.77	7.86

^*^The mean difference is significant at the 0.05 level.

**Table 4:** Scheffe test comparison of differences in domestic violence based on age.

(I) age	(J) Age	Mean difference (I-J)	Std. error	Sig.	95% Confidence interval	
					Lower bound	Upper bound
18–29 years	30-44 years	4.20^*^	0.531	0.000	2.89	5.50
	≥ 45 years	* 14.64^*^	0.589	0.000	13.19	16.09
30–44 years	18–29 years	* -4.20^*^	0.531	0.000	−5.50	−2.89
	≥ 45 years	* 10.44^*^	0.496	0.000	9.22	11.66
≥ 45 years	18-29 years	−14.64^*^	0.589	0.000	−16.09	−13.19
	30-44 years	−10.44^*^	0.496	0.000	−11.66	−9.22

^*^The mean difference is significant at the 0.05 level.

Table 4 shows statistically significant differences in domestic violence based on age. Specifically, those over 45 years experienced less severe domestic violence as compared to those aged 30–44 years and 18–29 years, respectively. This findings suggests that, as the victims’ age progressed, the lower was the severity of domestic violence they experienced.

Table 5 presents the results of the t-test that was conducted to analyze the differences in domestic violence scores based on participants’ job and health status. The results showed that employed women experienced less severe domestic violence as compared to those who were not employed, and women who had good health experienced less severe domestic violence as compared to their counterparts who reported being “sick.”

**Table 5:** *t*-test analysis of domestic violence by participants’ job and health status.

	*N*	*M*	SD	*T*
Job status				
Employed	235	19.21	3.73	8.63^*^
Not employed	152	23.55	4.90	
Health status				
Good	218	19.63	3.83	7.94
Sick	169	24.07	5.11	

^*^Statistically significant at *p* < 0.05 level.

Table 6 presents a linear regression analysis examining the relationship between domestic violence and anxiety, depression, and quality of life. Since all five demographic variables exhibited significant group differences in the bivariate analyses, they were included as control variables in the regression analysis.

**Table 6:** Linear regression analysis of the association of domestic violence with anxiety, depression, and quality of life.

Variables	Constant	*B*	*F*	*P*	Beta	*R*	*R* ^2^	Adjusted *R*^2^	*t*	*P*
Anxiety	4.946	0.677	719.523	0.001	0.855	0.855	0.732	0.731	26.824	0.001
Depression	11.886	0.281	0.297.995	0.001	0.728	0.728	0.630	0.628	17.26	0.001
Quality of life	112.762	−1.007	559.234	0.001	−0.824	0.824	0.679	0.679	−23.648	0.001

Educational level, age, job status, and health status were included as control variables.

The findings indicated that, in the present sample, domestic violence was a significant predictor of anxiety, depression, and quality of life, explaining 73, 63, and 67.9% of the variance in the three variables, respectively, when controlling for the influence of the demographic variables. The remaining variance could be ascribed to other factors that necessitate investigation in subsequent studies.

## Discussion

Violence against women is an important social problem. Women who have experienced domestic violence experience serious physical and psychological health problems. The results of the current study showed that majority of participants (63.30%) fell in the low severity category for domestic violence, whereas roughly one third (31.26%) reported moderate levels of domestic violence. A small portion (5.42%) reported experiencing severe domestic violence. Though the present study did not aim to determine the prevalence of domestic violence as the sample was restricted to women experiencing such abuse, the present dataset provides some insight on the prevalence of the types of domestic violence in this sample. Specifically, the incidence of moderate to severe levels of psychological abuse was 55.54%, followed by 40.82 and 13.4% for moderate to severe levels of physical and sexual abuse. In this regard, Eldoseri et al. (62) reported that 28% of the women in their sample experienced moderate to severe violence, with 29% reporting psychological abuse, 11.6% reporting physical abuse, and 4.8% reporting sexual abuse. In another study, the rate of domestic violence was 43.0%, and the most common type was dominant behavior (36.8%), followed by psychological abuse (22%), sexual abuse (12.7%), and physical abuse (9.0%) (48, 63). In general, the present sample seems to exhibit substantially higher incidence of the three types of domestic violence. However, Alquaiz et al. (48) and Alhabib et al. (63) emphasized that caution should be exercised when comparing the prevalence of domestic violence in different studies due to the lack of consistent definitions of domestic violence and/or its types. Methodological differences across studies conducted within the Kingdom of Saudi, such as differences in study period, design, target population, sampling methods, data collection methods, etc., could be one reason for this. Furthermore, social and cultural norms of each region in Saudi Arabia differ significantly (64). For instance, the southern region, where the present study was conducted, tends to be more traditionalistic, and therefore rendering the women residing in this region more vulnerable to the sociocultural factors and barriers that condone or perpetuate domestic violence against women (65). Interestingly, the present findings also suggested that all participants experienced the different types of domestic violence at least to some extent. However, this finding is not surprising because, in their studies on different types of domestic violence against women in Turkiye, Alkan et al. (15–17) reported that women who had experienced one type of domestic violence were more likely to experience other types as well.

The present study also found significant differences in domestic violence scores based on health status, age, educational level, and job status. Specifically, women who reported good health, older women, those with a higher educational level, and those who were employed tended to exhibit lower domestic violence scores as compared to their counterparts. With regard to the influence of health status, the present cross-sectional study cannot determine causality; therefore, it is unclear if the health problems contributed to the experience of abuse or if the abuse led to health problems. However, victims of domestic violence have been reported to experience health problems including from chronic diseases (high blood pressure and diabetes), gynecological problems (66), poor self-reported physical health, pain, injuries, and difficulties with daily activities (67).

Considering the other three demographic factors examined in the present study, similar insights have been drawn from other studies conducted in different context. For instance, among other factors, age, educational status, and income status were identified as vulnerability-inducing factors for women who experienced domestic violence in Turkiye (68) and North African and Middle Eastern countries (20). Similarly, using nationally representative data, Alkan, Bayhan (69) examined factors associated with different types of intimate partner violence experienced by women in Turkiye. In addition to past history of violence, partner’s alcoholism and gambling behaviors, etc., they found low income and educational levels to increase women’s risk for experiencing intimate partner violence. Kouyoumdjian et al. (70) reported that poorly educated women were more likely to experience domestic violence, while a Saudi study revealed that older women were less likely to report domestic violence (48).

Though the actual mechanisms of influence of women’s age, educational level, and employment status (and consequently their economic independence) have not been studied in-depth, one could conjecture that it may be linked to their social status, empowerment, awareness of rights, and access to help. This perspective is supported by Fageeh (25), who purported that the unemployed women in their sample from Jeddah, Saudi Arabia, were financially dependent on their husbands; therefore, they exhibited double the odds of experiencing physical aggression from their husbands. Similarly, Karakoc et al. (71) noted that women’s lack of economic freedom increases their risk of violence. Together, the current findings and the extant literature suggest that socioeconomic factors such as age, education, and employment/financial status could play a protective role against women’s vulnerability to domestic violence. This insight reveals the potential for focusing anti-domestic-violence interventions on empowering women with education and labor-force participation. However, women cannot be truly empowered until the underlying structural and systemic barriers at the familial and societal levels are not addressed. Indeed, the sociocultural factors influencing the experience of domestic violence are complex. For instance, from the 30 studies Mojahed et al. (72) included in their systematic review, it was evident that factors associated with higher levels of intimate partner violence among women in Arab countries included individual-, family-, community-, and societal-level influences. Similarly, a systematic review of 16 Saudi studies on intimate partner violence among women reported that a combination of personal, inter-personal, and sociocultural factors caused women’s vulnerability to experience of domestic violence by their partner (40). These reviews therefore recommend the urgent need for developing systematic and culturally sensitive interventions. Here, it is imperative to further understand the Saudi socio-cultural context.

Barnawi (44) suggested that, in the Saudi culture, owing to gender inequity and erroneous interpretations of Islamic religious tenets, both husbands and wives may believe that domestic violence toward the wife is a justified response to the wife’s misconduct. This view may be prevalent in the Southern region, which is characterized by preserving traditions, customs, and values and enjoying tribalism. Such communities also accept domestic violence, especially psychological or verbal violence, and justify it based on cultural and social norms (48, 73). Further, in the conservative culture, the shame or social stigma attached to reporting abuse, the normalization of some acts of abuse that perpetuate male dominance, the fear being severely abused if they do something to defy the perpetrator, and the lack of knowledge on how to seek or find help, may be prevalent (74). Other studies have confirmed these findings, with nearly 50% of women in the Middle East reporting that they tolerated domestic violence and were unable or unwilling to seek help from legal or social authorities or healthcare providers (42, 75). Although the Ministry of Social Affairs in Saudi Arabia has set up a hotline to help women and children experiencing domestic violence, the use and effectiveness of this service are questionable (44), largely reflecting a culture of tolerance, or disregard for violence within Saudi society. Furthermore, even when women may muster the courage to seek help, they may not have access to legal recourse owing to the gender-biased laws in Saudi Arabia (50).

Therefore, when considering ways to address and/or prevent domestic violence against women, empowerment-based interventions for victims are essential. Specifically, protection services and legislation should focus on a multi-factor approach to address the problem (48). A public health perspective could capture the many dimensions of the phenomenon to develop a better action plan based on evidence and best practices (2, 76). Women who have experienced domestic violence must also be seriously assessed in terms of mental health. In addition, counseling and psychotherapy services focused on domestic violence must be provided to the perpetrator and the entire family, including women. Healthcare physicians and health workers should have sufficient information about helping women who have experienced domestic violence develop a safety plan, inform them of their legal rights and the support they can get, protect them when necessary, and to provide support for mental health problems.

It is also important to study the characteristics and influencing factors from the perspective of the perpetrators of violence. For instance, Alkan et al. (69) reported that the husband’s educational level, gambling and alcoholism tendencies, and affinity to having violent outbursts in social settings also increased his wife’s vulnerability to experiencing violence at his hands. Therefore, interventions also need to focus on perpetrators of domestic violence, which have been reported to show consistently positive results in reducing the risk for intimate partner violence (77). However, a meta-analysis and systematic reviews of such interventions also confirmed the effectiveness of trauma-informed and substance abuse treatments in this regard (78). Similarly, as addressed earlier, the Saudi society is male dominant, with men enjoying the power in interpersonal relationships as well, including the marital dyad. This can be achieved by raising awareness, mentoring, and having open conversations about interpersonal dynamics in marital relationships, seeking professional help when problems occur, and not underestimating or ignoring the first stages of domestic violence. Relevant information may also be included in a course on marriage counseling for university students or a mandatory premarital course offered through mental health professionals or social/religious institutions.

Finally, interventions that focus on empowering women on one end and on addressing the power structures in familial, social, and politico-legal spheres on the other end need to be prioritized. These discussions need to be brought into the larger discourse in the academic and non-academic world; however, they are not elaborated further in this paper as the present study only considered the victim’s perspective. Future studies need to also examine the perpetrator’s viewpoint in Saudi Arabia.

Returning to a discussion of the present findings, the results of the regression analysis confirmed the association of exposure to domestic violence with mental health and quality of life outcomes. Existing studies have confirmed this association, highlighting the negative effects of domestic violence on the mental and physical health of women and their children and its long-term consequences, including mental and physical health problems (44, 48, 79). Women who have experienced domestic violence were more likely to experience poor mental health, long history of illness, miscarriage, and vaginal bleeding (42). Furthermore, the higher the levels of anxiety and depression are, the lower is the quality of life (80). Women who have experienced domestic violence are more likely to suffer from depression and severe anxiety symptoms, are three times more likely to contemplate suicide as compared to women who have not experienced domestic violence (71). Similarly, Ferrari, Agnew-Davies (32) reported that an increase in domestic violence is associated with worse mental health, especially anxiety, depression, and PTSD. Moreover, their study showed that domestic violence was significantly associated with lower quality of life in women who have experienced violence. In contrast, women who left their abusive partners exhibited an improvement in their quality of life (81). Again, in terms of interventions, as suggested by Sahebi, Golitaleb (82), it is crucial to consider the unique experiences and vulnerabilities of women while developing supports and interventions for addressing and preventing violence. For instance, considering the close association of domestic violence with mental health outcomes, interventions that focused on reducing the negative effects of violence by using therapeutic techniques such as Cognitive Behavioral Therapy (CBT) and those which seek to prevent revictimization by developing coping skills, have been found to be effective (77).

## Conclusion

The main objective of this study was to determine the nature of domestic violence experienced by women in Saudi Arabia and the extent to which demographic factors influenced domestic violence levels. It also aimed to determine the association of domestic violence with depression, anxiety, and quality of life among participants. The findings showed differences in the level of domestic violence based on participants’ age, educational level, job status, and health status. Specifically, the results showed that older women, those with higher levels of education, employed women, and those reporting good health tended to experience lower levels of domestic violence than their counterparts. Further, the current and past research provides strong evidence of the negative effects of domestic violence on the mental health and quality of life of women. Therefore, it is important to recognize this as a public health concern and develop appropriate strategies to prevent domestic violence and provide support to victims and perpetrators of such abuse considering their unique needs.

### Limitations of the study

Though the present study makes valuable contributions to the literature on women’s experience of domestic violence in Saudi Arabia, it has some limitations that need careful consideration when interpreting and generalizing these findings. The current study had several key sampling and data collection limitations. Specifically, using social networking sites to recruit participants could have led to selection bias, as only those with access to social media could respond. Therefore, study participants may not be representative of the wider population of women who have experienced domestic violence in Saudi Arabia. Moreover, the sample was not varied enough to reflect the social reality of the Kingdom of Saudi Arabia, such as women who do not meet one or more of the selection criteria specified for this study. It should also be considered that the sample was drawn from one province alone. As the sociocultural fabric of each province in Saudi Arabia is quite unique, this may limit the generalizability of the results to all Saudi women.

Furthermore, this study employed the cross-sectional design, which cannot determine causality when examining the association between key variables. These variables could have bidirectional causal relationships and/or have multiple intervening factors that mediate the association. Further, it utilized a relatively small sample. However, this sample size was estimated using the G*Power tool with predefined power and error limits. Therefore, the sample of 387 women was sufficient to derive robust statistical validity. Nevertheless, future studies should consider the use of different designs (e.g., longitudinal) and a larger, more diverse, and representative sample to provide a clearer picture of the general population. Finally, the study excluded the male viewpoint by only including female participants. Future comparative studies should include males and females to provide a more comprehensive understanding of the studied variables.

## Data Availability

The original contributions presented in the study are included in the article/supplementary material, further inquiries can be directed to the corresponding authors.
